# Intraoperative Neurophysiological Monitoring in Full-Endoscopic Cervical Endoscopic ULBD

**DOI:** 10.3390/jcm15010327

**Published:** 2026-01-01

**Authors:** Miles Hudson, Sarah Esposito, Mark M. Zaki, Simon M. Glynn, Osama N. Kashlan, John Ogunlade, Chandan Krishna, Joshua Bakhsheshian, Christoph P. Hofstetter

**Affiliations:** 1 Mayo Clinic Department of Neurosurgery, 5777 E Mayo Blvd, Phoenix, AZ 85085, USA; 2 Department of Neurosurgery, University of Michigan, 1500 E Medical Center Dr, Ann Arbor, MI 48109, USA; 3 Taylor Family Department of Neurosurgery, Washington University, St. Louis, MO 63110, USA; 4 Department of Neurosurgery, University of Washington, 908 Jefferson St, Seattle, WA 98104, USA; chh9045@uw.edu

**Keywords:** endoscopic, spine, transforaminal, cervical, cervical myelopathy, spinal stenosis, motor evoked potentials, surgical complications

## Abstract

**Background/Objectives:** To evaluate risk factors for postoperative neurological deficits following cervical endoscopic unilateral laminotomy for bilateral decompression (CE-ULBD) and to determine whether intraoperative neurophysiological monitoring (IONM) can predict neurological compromise. **Methods:** A multicenter retrospective review was performed on 42 CE-ULBD procedures conducted between 2016 and 2024; 33 cases met the inclusion criteria with available imaging and electromyography data. Demographic, operative, and neurophysiological variables were analyzed. Preoperative stenosis severity was graded using the Kang MRI system. Intraoperative IONM data, including electromyography firing and motor evoked potential (MEP) changes, were correlated with new postoperative weakness. **Results:** The cohort (69.1% male, mean age 70.2 ± 1.7 years, mean BMI 29.6 ± 1.1) included 56 decompressed levels. The most common operative levels were C3-4 (37%) and C4-5 (24%). Postoperative weakness occurred in four patients (12.1%), all of whom had severe (Grade 3) preoperative stenosis. Among these, 50% exhibited preoperative weakness. Neuromonitoring changes correlated significantly with postoperative weakness (Fisher’s Exact, *p* < 0.001); 100% of patients with new post-operative weakness had sustained MEP decrease at the time of closure. **Conclusions:** Patients with severe cervical stenosis and preoperative weakness are at heightened risk of postoperative neurological deficits following CE-ULBD. Elevated epidural pressure from continuous irrigation in a constricted canal may exacerbate cord compression, particularly in those with preexisting myelopathy. IONM changes strongly correlate with new deficits and may exacerbate cord compression, particularly in those with preexisting myelopathy, and may serve as an early warning system for impending neurological injury. Surgeons should exercise caution and maintain low irrigation pressures in patients with severe stenosis undergoing endoscopic cervical decompression.

## 1. Introduction

Over the past few decades, technological innovations, expanded training opportunities, and growing patient demand have driven the broader adoption of both instrumented and non-instrumented minimally invasive spine surgery. Full-endoscopic spine surgery is one of the latest advancements in minimally invasive tubular spine surgery [1]. Utilizing an endoscope with an integrated working channel, illumination, and irrigation, full-endoscopic spine surgery—originally developed for the transforaminal approach [2]—has been adapted to address a wide variety of spinal pathologies [3]. Technical advancements, including irrigation pumps, high-speed burs, radiofrequency probes, and specialized surgical tools, have enabled the adaptation of the full-endoscopic technique for the interlaminar approach [4,5].

Several studies have demonstrated the efficacy of full-endoscopic decompression techniques in the lumbar spine [6,7,8]. Learning full-endoscopic techniques for the cervical spine has been shown to involve a steep but safe learning curve for surgeons experienced in lumbar endoscopic procedures [9]. The surgical technique of cervical endoscopic unilateral laminotomy for bilateral decompression (CE-ULBD) has been described in detail [10]. In this case series, we demonstrated the safety and efficacy of this procedure, which resulted in improved neurological function, as evidenced by significant improvements in Nurick grade and mJOA scores. More recent data suggest that patients experience relief from neck and arm pain 90 days after surgery and experience favorable physical activity patterns following decompression of the cervical spinal cord [11]. Complication rates following CE-ULBD have been reported to range from 1.15% to 33.3% [12]. Reported complications include neurological deficits such as motor weakness or hypesthesia, persistent postoperative pain, and intraoperative durotomy [12]. Although patient-specific risk factors for complications in CE-ULBD have not been thoroughly investigated, optimal training, the use of specialized instruments, and meticulous operative technique are essential for minimizing adverse outcomes in full-endoscopic spine surgery [12,13]. This study aims to identify risk factors for postoperative neurological symptoms following CE-ULBD and to assess whether intraoperative neurophysiological monitoring can detect and alert surgeons to early signs of impending neurological compromise.

## 2. Materials and Methods

### 2.1. Study Design

Retrospective review and analysis of cervical endoscopic unilateral approaches for bilateral decompression (CE-ULBD), completed at the University of Washington, Washington university, and the University of Michigan between 2016–2024, was performed. The inclusion criteria included patients who underwent CE-ULBD at the institutions between 2016 and 2024. The exclusion criteria included patients without neuromonitoring data, without imaging to review, and patients who underwent additional operations other than CE-ULBD at the time of the index surgery. This review illustrated 42 operative cases; of those cases, 9 were excluded due to a lack of magnetic resonance imaging to review or a lack of electromyography data. In total, 33 cases were thereby included in this study.

### 2.2. Retrospective Evaluation

Patient charts were reviewed for demographic, clinical, and operative data. New post-operative weakness was classified as immediate post-operative weakness with any strength deficit lower than their baseline exam, continued at 2-week follow-up. For the assessment, a Manual Muscle Test (MMT) was performed, with new postoperative weakness defined as a decrease of ≥1 grade or subgrade in any tested myotome compared with the preoperative exam. Patient preoperative MRI imaging was analyzed, and the degree of central stenosis was recorded for each patient based on the schema described by Kang et al. [14] (Table 1). Patients in the grade 0 category had no stenosis, whereas grades 1, 2, and 3 had mild, moderate, and severe, respectively. All MRI scans were evaluated on T2-weighted sagittal and axial sequences using the Kang grading system. The highest grade at the operative level was recorded for analysis in patients with multilevel disease.

### 2.3. Surgical Technique

Under general anesthesia, patients were positioned prone on a Jackson bed with the head secured in a Mayfield^®^ head holder. All patients underwent neurophysiological monitoring throughout the case, including electromyography (EMG) and motor-evoked potentials (MEPs), and the irrigation pressure was maintained at 37 mmHg (50 cm H_2_O). As previously described (REF Carr et al. 2020 [10] Hofstetter Thieme book), anteroposterior and lateral fluoroscopic guidance was used to identify the appropriate level. Following infiltration with local anesthetic, a vertical skin incision was performed with an #11 blade. Using a Seldinger-style technique, serial dilators were carefully advanced over a guidewire and docked onto the adjacent laminae at the index level. A tubular retractor with a 30-degree bevel was placed to provide access for a working-channel endoscope (iLESSYS^®^ Pro, joimax^®^ Inc., Irvine, CA, USA). The laminotomy was performed along the insertion of the yellow ligament with a 3.5 mm diameter diamond burr. The ligamentum flavum was resected with Kerrison rongeurs and micro-punches. For safe over-the-top decompression of the contralateral side, the juxtaposed aspects of spinous processes were generously undercut using the diamond burr. The contralateral lamina was then undercut with a combination burr, Kerrison, and micro-punch.

### 2.4. Neuromonitoring Reports

Patient neuromonitoring reports were reviewed for changes throughout the procedure. Reports were reviewed for any changes to the motor potentials, including EMG firing, temporary motor decrease, or sustained motor decrease, that persisted at the time of closure. A motor decease was defined as >50% decrease in monitoring potentials.

### 2.5. Statistical Analysis

Statistical comparisons were performed using Fisher’s Exact Test for categorical variables, with significance defined as *p* < 0.05.

## 3. Results

Our study cohort included 33 patients, of which 69.7% were men (Table 2). The average age of patients included was 70.2 ± 1.7 and the average BMI was 29.6 ± 1.1. Preoperative motor weakness was present in 60.6% of operative cases.

All patients tolerated the procedures well. In total, 16 patients underwent decompression of the cervical spinal cord at a single level, 14 patients had two operative levels, 2 patients had three operative levels, and one patient had a 4-level operation (Table 3). In total, 54 levels were included in the CE-ULBD cohort. The most common operative level was at C3-C4, with 20 interventions at this level, followed by C4-C5 with 13 interventions. CE-ULBD took on average 91.8 min per level, with an average blood loss of 9.5 mL. Post-procedural weakness, which was new at onset, was recorded in four of the 33-patient cohort. In these patients, the average time per level, measured in minutes, was 75 min, with an average blood loss of 6.75 mL compared to a mean operative time of 94.1 min in patients without new deficit. Severe central stenosis (Grade 3) was the most common category, present in 21 of 33 patients (63.6%).

The pre-operative degree of spinal stenosis at the index level was determined according to the Kang grading scale [14] (Table 4). Our cohort had 1 patient with a mild degree of stenosis (grade 1) and 11 with grade 2. Severe grade-3 stenosis was the most common occurrence in 21/33 patients in our cohort. Novel postoperative weakness was only encountered in patients who had preoperative grade 3 stenosis at the index level. In patients with grade 3 stenosis, 4/21 (19%) had new post-operative weakness.

Manual muscle exam testing was performed on all patients pre and post-operatively. Four patients (12.1%) had a decrease of ≥1 grade or subgrade in any tested myotome compared with the preoperative exam at two-week follow-up (Table 5). Two out of four (50%) patients with new post-operative weakness had associated pre-operative weakness.

The EMG results for patients with any intraoperative changes or post-operative weakness are seen categorized in Table 6. For patients with intra-operative neuromonitoring changes, new post-operative weakness was only observed in patients with sustained MEP decrease at the time of closure. Four out of five patients with sustained MEP decrease at the time of closure had new post-operative weakness (80%). A Fisher’s Exact test was performed, demonstrating that EMG changes from baseline were associated with new post-operative weakness (*p* < 0.001). In total, 100% of the patients who presented with post-operative weakness also had at least one level of Grade 3 stenosis.

## 4. Discussion

Endoscopic approaches to the cervical spine, including foramino-discectomies, laminectomies, and laminotomies, have been well described in the literature [15,16,17,18,19]. The procedure has been documented to be safe and effective for treating cervical pathologies, with reported complication rates being comparable to anterior cervical approaches [20]. Though complication rates remain low, endoscopic approaches to the cervical spine can carry a considerable risk of neurologic injury due to the nature of the approach itself, the presence of cord containing levels, and the irrigation pressure required during endoscopy. The risk profile continues to be studied, but one such area lacking data is the ramifications of the continuous irrigation pressure and its effects on the spinal system. When performing these procedures, the irrigation pressure should be less than the patient’s diastolic blood pressure to prevent venous congestion, with ideal values ranging from 20–40 mm Hg [15,21,22]. In a study performed by Choi et al. [23], cervical epidural pressure (CEP) and neck pain were analyzed in patients undergoing percutaneous endoscopic lumbar discectomies (PELD). Participants had a mean pre-operative CEP of 15.5 mm Hg compared to a maximum pressure of 71 mmHg in patients with neck pain and 44.3 mmHg in patients without neck pain. These results indicate that cervical endoscopic approaches to the spine may significantly increase epidural pressures. The mechanism proposed involved intracanalicular irrigation filling the epidural space, increasing pressure and compressing the thecal sac. In this study, we performed a retrospective review to determine if there was any correlation between the degree of pre-operative cervical stenosis and the risk of post-operative neurologic deficit.

Thirty-four patients were reviewed in this study, comprising a total of 54 operative levels. Within this cohort, we had four patients with new post-operative deficits (12.1%). Of this group, 100% presented with sustained motor-evoked potential decrease at the time of closure. Further review demonstrated that 100% of these patients with post-operative weakness had at least one operative level consistent with grade 3 (severe) stenosis and cord signal change. Intra-operative blood loss was minimal for all the procedures and there was no significant difference in the average operative time per level at 75 min per level in the group with new post-op weakness and 94.1 min per level in the group without new weakness. Two out of four (50%) of the patients with new post-op weakness also had associated pre-operative weakness. The overall complication rate and outcomes contrast with the reported literature, where overall complication rates range from 0–10% [15,24,25], with minimal reports of post-op weakness (0–2%). These results contrast with our much higher rates of post-operative weakness (12%). These results may be due to the high percentage of patients in this study who had severe grade 3 stenosis and pre-operative deficit. When comparing these results to the current literature, many studies did not comment on the degree of pre-op stenosis and/or excluded patients with severe cervical stenosis, limiting the comparative ability of our study. This data seems to demonstrate that patients with a high degree of cervical stenosis and pre-operative weakness are at a higher risk of post-operative neurologic weakness.

Technical factors may also play a role in the complication factors of this surgery. The procedures were completed by experienced surgeons, but the intra-operative time is a consideration and was not insignificant in either group, at 94.1 min per level in patients without new weakness and 75 min per level in patients with new weakness. In the cervical spine, the risks of weakness due to direct/indirect trauma of the cord were significantly higher in an already narrowed canal. The high complication rate in our series may reflect a combination of a high-risk patient population and the intrinsic challenges of a technically demanding procedure, especially in its early adoption phase at the participating center.

**Figure 1:**
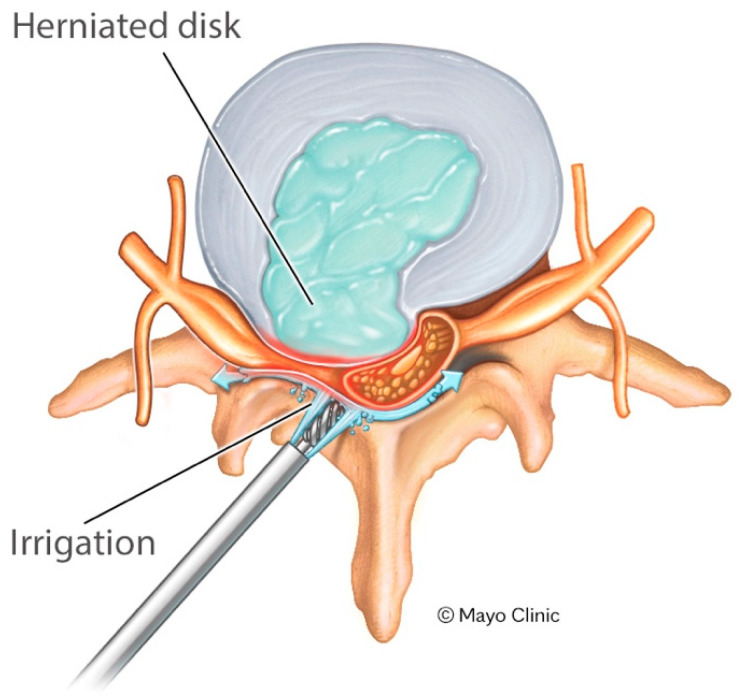
Proposed mechanism for possible increased stress and epidural pressure on the Cervical Spinal cord due to irrigation pressure.

The association between preoperative myelomalacia and increased susceptibility to postoperative neurological decline has been shown in the literature. Prior studies have demonstrated that intramedullary T2 signal change is correlated with reduced postoperative recovery, and that the extent and severity of such signal abnormalities predict poorer neurological outcomes following cervical decompression. Collectively, these findings suggest that chronically compressed or injured spinal cords possess diminished physiological reserve and may tolerate mechanical stress, traction, or transient perfusion shifts during surgery less effectively than unaffected cords [26,27]. We also propose an additional contributing mechanism for the increased rates of post-operative weakness. Though the data generated by Choi et al. [23] demonstrated increased CEP during endoscopic lumbar surgery, we propose that the endoscopic irrigation pressure during endoscopic cervical surgery would have the same effect and may also increase CEP during an endoscopic approach. This effect may be compounded in the cervical spine in cases of severe pathology, as anterior compression occurring in severe disease may pincer the cord and further worsen the effects (Figure 1). The neurologic sequelae are likely to be more profound in the cervical spine as there is cord instead of nerve roots and patients who already have cord injury, demonstrated by cord signal change, may be pre-disposed to further damage due to prior injury and limited reserve.

This study does have weaknesses, including a limited cohort size and a cohort with new weakness occurring in patients with both severe central and pre-operative weakness. Further studies and evaluation are needed to fully delineate the risks involved in endoscopic approaches to the cervical spine in patients with high-grade cervical stenosis.

Larger, prospective studies are needed to confirm the association between severe preoperative stenosis, preoperative weakness, and postoperative neurological decline following CE-ULBD. Future work should focus on refining patient selection criteria, particularly for individuals with grade 3 stenosis, to better define when endoscopic decompression may carry elevated risk.

Further investigation into the role of irrigation-related epidural pressure is warranted. Direct intraoperative measurement of pressure dynamics, integrated with real-time IONM data, may clarify whether heightened canal pressure contributes to neurological compromise and could guide the development of intraoperative thresholds for modifying irrigation flow or surgical technique. Technical refinements also merit exploration, including lower irrigation pressures, staged decompression strategies, or alternative minimally invasive approaches for patients with limited canal reserve.

## 5. Conclusions

Endoscopic approaches to the cervical spine are an invaluable tool that can achieve post-operative outcomes on par with open approaches resulting in a reduced length of stay and faster recovery. This study demonstrates that intra-operative neuromonitoring changes correlated with new post-operative weakness. Patients with pre-operative weakness and a high degree of cervical central may be at a higher risk of neurologic deficit post-operatively. During endoscopic procedures, cumulative forces applied to the cervical cord may be affected by the tight corridor and limited epidural space in cases of severe stenosis further affected by irrigation pressure. This may result in a higher risk of neurologic injury and post-operative weakness in this patient population.

## Figures and Tables

**Table 1:** Kang grading system for central cervical stenosis.

Grade 0	No Stenosis	
Grade 1	Mild Stenosis	>50% thecal Sac compression without cord deformity
Grade 2	Moderate Stenosis	Cord Deformity without signal Change
Grade 3	Severe Stenosis	Cord Signal Change

**Table 2:** Patient demographics.

Demographic	Result
Male	23 (69.7%)
Female	10 (30.3%)
Mean Age (years)	
Male	69.13 ± 2.1
Female	72.5 ± 2.9
BMI Mean	29.6 ± 1.1
Primary Pre-op Diagnosis	
Cervical Stenosis	33
Preoperative Weakness	
Yes	20 (60.6%)
No	13 (39.4%)

**Table 3:** Operative data.

Operative Data	Result
Number of Operative Levels	
One	16
Two	14
Three	2
Four	1
Average Time per level (min)	91.8
Average Blood Loss (mL)	9.5
CE-ULBD Procedural Levels	
C2-C3	2
C3-C4	20
C4-C5	13
C5-C6	9
C6-C7	10
Total	54
New Post-Procedural Weakness	
Yes	4 (12.1%)
Mean blood loss (mL)	6.75
Time per Level (min)	75
No	28 (82.4%)
Mean blood loss (mL)	9.9
Time per level (min)	94.1

**Table 4:** Central stenosis grades.

	Highest Degree of Stenosis Per Patient	Patients With New Post-Op Weakness
Central Stenosis		
Grade 1	1	0
Grade 2	11	0
Grade 3	21	4 (19%)

**Table 5:** Manual muscle exam testing results pre/post-operatively for patients with new post-operative weakness.

Surgical Levels	Pre-Operative Weakness	Stenosis Grade	Pre-Operative Exam	Two-Week Post-Operative Exam
C3-C5	No	3	Intact	Right: 4+/5 Bicep, 4+/5 Triceps, Remainder Intact Left: Intact
C3-C4	Yes	3	3+/5 Bilateral Upper extremities 4/5 bilateral dorsiflexion, plantar flexion, knee hip flexion Left Knee flexion 3+/5, Right Knee flexion 4/5	Right: 2/5 deltoids, 3/5 triceps, 3+/5 biceps, 0/5 intrinsics, 0/5 grip. 3+/5 dorsiflexion, 4/5 knee flexion, knee extension and hip flexion. Left: 3+/5 deltoids, 3+/5 triceps, 3+/5 biceps, 3+/5 intrinsics, 2/5 grip. 4/5 in left dorsiflexion. 4/5 knee flexion, knee extension and hip flexion
C3-C5	No	3	Intact	Right: Intact Left 4+/5 deltoid, Remainder of exam intact
C3-C4	Yes	3	Right: Bicep 4/5, Triceps 4/5, Deltoid 4/5, 4+/5 Hand intrinsics, Remainder of exam intact Left: 5/5 except Hand intrinsics 3/5	Right: Bicep 4/5, Triceps 3/5, Deltoid 2/5. Wrist extension 5/5, Flexor Digitorum Profundus 4/5, Abductor Digiti Minimi 2/5, Remainder of exam intact Left: Bicep 2/5, Triceps 2/5, Deltoid 2/5, Wrist extension 2/5, Flexor Digitorum Profundus 4/5, Abductor Digiti Minimi 2/5, Remainder of exam intact

**Table 6:** Neuromonitoring results in patients with and without new-post-op weakness.

Procedure	Procedural Level	Pre-Op Weakness	Highest Stenosis Grade	Intra-Op IONM Results	New Post-Op Weakness
CE-ULBD	C3-C4	Yes	3	Sustained MEP Decreased	Yes
CE-ULBD	C3-C5	No	3	Sustained MEP Decrease	Yes
CE-ULBD	C3-C5	No	3	Sustained MEP Decrease	Yes
CE-ULBD	C3-C4	Yes	3	Sustained MEP Decrease	Yes
CE-ULBD	C4-C5	No	3	Sustained MEP-Decrease	No
CE-ULBD	C4-C5	Yes	3	Temporary MEP Decrease	No
CE-ULBD	C3-C5	Yes	3	EMG Firing	No
CE-ULBD	C3-C5	Yes	3	EMG Firing	No
CE-ULBD	C2-C3	No	2	EMG Firing	No
CE-ULBD	C3-C5	Yes	2	EMG Firing	No
CE-ULBD	C5-C7	Yes	2	EMG Firing	No
CE-ULBD	C5-C7	Yes	3	EMG Firing	No
CE-ULBD	C3-C4	Yes	3	EMG Firing	No
CE-ULBD	C6-C7	No	3	EMG Firing	No
CE-ULBD	C3-C4	Yes	1	EMG Firing	No
CE-ULBD	C4-C7	No	2	EMG Firing	No
CE-ULBD	C4-C6	No	3	EMG Firing	No
CE-ULBD	C3-C6	No	2	EMG Firing	No
CE-ULBD	C5-C7	Yes	2	EMG Firing	No
CE-ULBD	C3-C4, C6-C7	No	2	EMG Firing	No
CE-ULBD	C5-C7	Yes	3	EMG Firing	No

## Data Availability

The datasets generated and/or analyzed during the current study contain protected health information (PHI) and are therefore not publicly available. Access to these data may be granted upon reasonable request and with appropriate permissions, in accordance with institutional and ethical guidelines.

## Abbreviations

The following abbreviations are used in this manuscript:

CE-ULBD	Cervical Endoscopic Unilateral Laminotomy for Bilateral Decompression
IONM	Intraoperative Neurophysiological Monitoring
EMG	Electromyography
MEP	Motor Evoked Potential
MRI	Magnetic Resonance Imaging
BMI	Body Mass Index
mJOA	Modified Japanese Orthopedic Association (score)
